# Perinatal outcomes and sinopharm BBIBP-CorV vaccination during pregnancy

**DOI:** 10.1186/s12884-024-06389-z

**Published:** 2024-03-11

**Authors:** Donya Hatami, Abbas Habibelahi, Nasrin Changizi, Mohammad Heidarzadeh, Marzieh Nojomi, Moein Rast, Kiarash Ansari, Arash Tehrani‐Banihashemi

**Affiliations:** 1 Preventive Medicine and Public Health Research Center, Psychosocial Health Research Institute, Department of Community and Family Medicine, School of Medicine, Iran University of Medical Sciences, Tehran, Iran; 2 Neonatal Health Office, Ministry of Health and Medical Education, Tehran, Iran; 3 Maternal Fetal and Neonatal Research Center, Tehran University of Medical Sciences, Tehran, Iran; 4 Department of Neonatology, Zahedan University of Medical Sciences, Zahedan, Iran

**Keywords:** COVID-19, COVID-19 vaccines, Perinatal outcome, Maternal COVID-19, BIBP COVID-19 vaccine, Vaccine safety

## Abstract

**Background:**

After the outbreak of COVID-19, a huge part of the health care services was dedicated to preventing and treating this disease. In case of COVID-19 infection, severe COVID-19 is reported more in pregnant individuals. Afterward, Vaccination against SARS-CoV-2 became a hot topic due to known effects in preventing severe COVID-19 during pregnancy.

Vaccination of pregnant individuals started in August 2021 with the Sinopharm vaccine in Iran. The aim of current study was to determine the incidence of perinatal outcomes in women who were vaccinated during pregnancy.

**Method:**

This retrospective cohort study included 129,488 singleton births from March 21, 2021, until March 21, 2022, in Tehran, Iran.

The data was obtained from the Iranian Maternal and Neonatal (IMaN) Network and the Maternal Vaccination Registry.

Adverse perinatal outcomes investigated in this study include preterm birth, extremely preterm birth, low birth weight, very low birth weight, intrauterine growth restriction, stillbirth, neonatal intensive care unit admission, congenital anomaly, neonatal death and low 5-minute Apgar score. The risk of all perinatal outcomes was evaluated using multiple logistic regression. The analysis was done using STATA version 14.

**Results:**

Of all 129,488 singleton births included in this study, 17,485 (13.5%) were vaccinated against SARS-CoV-2 (all with Sinopharm (BBIBP-CorV)). The exposure to the Sinopharm vaccine during pregnancy caused a significant decrease in the incidence of preterm birth (*P* =0.006, OR=0.91 [95% CI, 0.85 to 0.97]), extremely preterm birth (*P* =<0.001,OR=0.55 [95% CI, 0.45 to 0.66]), and stillbirth (*P* =<0.001, OR=0.60 [95% CI, 0.47 to 0.76]). Exposure to vaccination during the first trimester was associated with an increased risk of preterm birth (*P* =0.01, OR=1.27 [95% CI, 1.04 to 1.55])

Maternal vaccination during pregnancy was not associated with an increased risk of other adverse perinatal outcomes included in this study.

**Conclusion:**

The finding of this population-based study indicated no adverse pregnancy outcome due to vaccination with the Sinopharm vaccine during the second and third trimesters of pregnancy. Overall risk of adverse pregnancy outcomes were lower in the vaccinated individuals compared to the unvaccinated group. Also, vaccination during the first trimester was associated with an increased risk of preterm birth.

## Background

After the outbreak of COVID-19 in December 2019, a huge part of the health care services was dedicated to preventing and treating this disease. In the case of COVID-19 infection, hospitalization and intensive care unit admission are reported more in pregnant individuals [1, 2]. Also, perinatal outcomes such as preterm birth, especially by cesarean section, low 5-minute Apgar score, admission to neonatal intensive care unit, and neonatal death was more common in newborns who were born from mothers infected with COVID-19 during pregnancy [3]. IRAN had one of highest rates of death due to COVID-19 in Eastern Mediterranean Region [4]. Vaccination against SARS-CoV-2 during pregnancy is recognized as a key factor in preventing maternal illness [5]. Nonetheless, the International Federation of Gynecology and Obstetrics and the US Centers for Disease Control and Prevention have since advised unrestricted COVID-19 vaccination in women currently pregnant [6, 7].

Sinopharm (BBIBP‐CorV) is an inactivated virus vaccine with an aluminum hydroxide adjuvant. Although the platform seems safe during pregnancy, the Food and Drug Administration has not yet approved aluminum hydroxide for usage during pregnancy due to the lack of data [8, 9]. The use of the Sinopharm vaccine against COVID-19 has received an interim recommendation from the World Health Organization for emergency use [10]. Sinopharm vaccination in general population seems to be safe with mild and predictable side effects like injection site pain, fatigue, headache and lethargy [11, 12]. For usage during pregnancy, "WHO recommends the use of COVID-19 vaccine Sinopharm in pregnant women when the benefit of vaccination outweighs the potential risks". However, the available information on using the Sinopharm vaccine in pregnant women is insufficient for determining pregnancy-related hazards [13]. After all, due to concerns about the safety of vaccination during pregnancy and lack of evidence, some people may be reluctant to get vaccinated while pregnant [14].

Almost all previous studies about adverse perinatal outcomes were done on mRNA vaccines like Pfizer-BioNTech (BNT162b2) and Moderna (mRNA-1273) or viral vector vaccines like AstraZeneca (AZD1222) [15–17].

Vaccination of pregnant individuals started in August 2021 with the Sinopharm vaccine in Iran. In this study, the risk of unfavorable perinatal outcomes following SARS-CoV-2 vaccination during pregnancy was examined using data from the maternal vaccination registry and Iranian Maternal and Neonatal (IMaN) Network.

## Method

### Sample and design

This retrospective-cohort study included all singleton births from March 21, 2021, until March 21, 2022, in Tehran, Iran. Information about maternal age and education, previous material conditions and background characteristics, and perinatal outcomes was obtained from the Iranian Maternal and Neonatal (IMaN) Network. The Maternal Vaccination Registry provided information on vaccinated pregnant individuals, including gestational age while vaccinated and vaccination dose. Women who received at least one dose of the Sinopharm vaccine between conception and childbirth were considered vaccinated in this study. The general recommendation for vaccinating pregnant women in Iran was made in August 2021 with the Sinopharm vaccine (BBIBP‐CorV). Routine vaccination was recommended for all pregnant individuals.

The mother’s national identification number was used to merge information gained by two datasets. In the study period, 135,272 births happened in Tehran, Iran. Of these, 5784 births were excluded after ending in multiple gestation. Therefore, 129,488 birth data were available for analysis.

## Perinatal outcomes

Adverse perinatal outcomes compared between vaccinated and unvaccinated individuals against SARS-CoV-2 included preterm birth (<37 completed gestational weeks), extremely preterm birth (<32 completed gestational weeks), low birth weight (<2500 gr), very low birth weight (<1500 gr), Intrauterine growth restriction [18], stillbirth, neonatal death (death of liveborn infant within first 28 days of life), congenital anomalies, low Apgar score (5-minute Apgar score<7) and neonatal intensive care unit admission.

Congenital malformations were defined as the presence of one or more congenital anomalies in examination at birth. The congenital anomalies in the Iranian Maternal and Neonatal Network included any malformation in the central nervous systems, musculoskeletal, gastrointestinal tract, cardiovascular, genitourinary, respiratory systems, eye-face-ear and skin.

### Measured predictors

Incidence of all perinatal outcomes was adjusted for maternal age (continuous), nulliparity (yes or no), mother's educational level (<=9 years, between 10 and 12 years, and more than 12 years), prepregnancy body mass index (less than 18.5, between 18.5 and 24.9, between 25 and 29.9, and more than 30), smoking or other substance use during pregnancy (yes or no) and maternal underlying medical conditions (yes or no).

Chronic hypertension or hypertensive disorders during pregnancy (including eclampsia or preeclampsia) [19], cardiovascular disease, diabetes or gestational diabetes were included as the maternal underlying conditions.

We adjusted low birth weight, very low birth weight, low Apgar score, neonatal intensive care unit admission and neonatal death for gestational age at birth as an additional covariant.

### Statistical analysis

Univariate logistic regression was used to obtain an unadjusted odds ratio for all perinatal outcomes.

The main goal of the study was to compare the incidence of perinatal outcomes in exposure to vaccination with the Sinopharm vaccine during pregnancy. The sub-goal of this study was the evaluation of any differences in risk of adverse perinatal outcomes according to the pregnancy trimester of vaccination or vaccine dose. Vaccination in different trimesters was defined as receiving the first vaccine dose during the first (<=13 +6 gestational week), second (14+0 to 27+6 gestational weeks) or third trimester (>=28+0 gestational weeks) of pregnancy.

Adjusted odds ratio was obtained using multiple logistic regression. The incidence of all outcomes was adjusted for all predictors. There are some missing data on pregnancy BMI and maternal educational level. By default in logistic regression, the analyzing software (STATA) uses listwise deletion of observations with missing values in any of the predictors.

Univariate logistic regression was used to compare categorical characteristic features in vaccinated and unvaccinated individuals. Also, we used t-test for comparing maternal age in these two groups.

Level of significance was considered at 0.05. Analyses were conducted using Stata version 14 (StataCorp).

## Results

Of all 129,488 singleton births included in this study, 17,485 were among mothers vaccinated against SARS-CoV-2 during pregnancy. In total, 6774(38.74% of vaccinated mothers against SARS-CoV-2) individuals received 1 dose of the Sinopharm vaccine during pregnancy whereas 10711(61.26%) had 2 doses while pregnant. The median gestational age at which vaccinated mothers received their first dose of the Sinopharm vaccine was 24 Weeks (IQR 19-29). This number was 26 Gestational weeks (IQR 21-31) for the second dose.

Among those vaccinated against SARS-CoV-2, 1202 (6.87% of those vaccinated) were vaccinated during the first trimester, 10250 (58.62%) were vaccinated during the second trimester, and 6033 (34.51%) were vaccinated during the third trimester.

Individuals vaccinated during pregnancy were generally multiparous (61.41% of vaccinated vs 54.56% of unvaccinated individuals). They were more likely to have underlying medical conditions (15.46% of vaccinated vs 13.53% of unvaccinated individuals) and less likely to be smokers or substance abusers (0.05% of vaccinated vs 0.22% of unvaccinated individuals). Individuals with a body mass index over 30 are more likely to receive a Sinopharm vaccine compared to the baseline group (individuals with a body mass index between 18.5 to 24.9). Whereas individuals with a body mass index less than 18.5 are less likely to be vaccinated. Vaccinated individuals were usually less educated (Table 1).

**Table 1:** Demographic characteristics of study population based on vaccination history

**Background characteristic**	**No.(%)** **Vaccinated (*n*=17,485)**	**Unvaccinated (*n*=112,003)**	***P* value**
Mother’s age ,mean (SD),y	31.33(5.75)	31.16(5.67)	<0.001
multiparous	10,737( 61.41 )	61,107(54.56)	Baseline
nulliparous	6,748(38.59)	50,896(45.44)	<0.001
Educational level ,y	17,304	111,152	
≤9	3,169(18.31)	12,284( 11.05)	Baseline
10-12	6,972(40.29)	44,176(39.74)	<0.001
>12	7,163(41.40)	54,692(49.20)	<0.001
pre pregnancy body mass index,	15,954	101,813	
18.5>	133(0.83)	1,607(1.58)	<0.001
18.5-24.9	11,900(74.59)	76,158(74.80)	Baseline
25-29.9	3,112(19.51)	19,624(19.27)	0.49
>30	809(5.07)	4,424(4.35)	<0.001
Maternal underlying medical conditions	2,696(15.46)	15,121(13.53)	<0.001
Chronic hypertension	361(2.07)	1,953(1.75)	0.003
Preeclampsia/ eclampsia	432(2.48)	2,557(2.29)	0.12
diabetes	140(0.80)	689(0.62)	0.004
Gestational diabetes	1,881(10.78)	10,293(9.21)	<0.001
Cardiovascular disease	105(0.60)	815(0.73)	0.06
Smoking or other addictions	9(0.05)	250(0.22)	<0.001

Of all births, 8.15% lead to preterm birth (7.98% of vaccinated vs 8.17% of unvaccinated individuals). After adjusted analysis, we observed a statistically significant decrease in the incidence of preterm birth in individuals vaccinated against SARS-CoV-2 during pregnancy (*P* =0.006, aOR=0.91, [95% CI, 0.85 to 0.97]) (Table 2). Comparing vaccination doses during pregnancy, getting 1 dose of Sinopharm vaccine led to no difference in risk of preterm birth. However, receiving 2 doses was associated with a decrease in incidence of this outcome (Table 3). Adjusted analysis showed that the first-trimester vaccination caused an increase in the incidence of preterm birth. There was no change in the incidence of preterm birth in those vaccinated during the second trimester. However, third-trimester vaccination caused a decrease in the incidence of preterm birth (Table 4).

**Table 2:** Crude and adjusted odds ratios of adverse *perinatal outcomes *according to COVID-19 vaccination

**Adverse pregnancy outcome**	**Vaccination status**	**No.**	**No of cases. (%)**	**Unadjusted odds ratio (95%CI)**	**Unadjusted *P* value**	**adjusted odds ratio (95%CI)**^a^	**Adjusted *p* value**
Preterm birth	unvaccinated	112,003	9,151(8.17)		Baseline		
	vaccinated	17,485	1,396(7.98)	0.97(0.91 to 1.03)	0.40	0.91(0.85 to 0.97)	0.006
Extremely preterm birth	unvaccinated	112,003	1,497(1.34)		Baseline		
	vaccinated	17,485	133(0.76)	0.56(0.47 to 0.67)	<0.001	0.55(0.45 to 0.66)	<0.001
Congenital anomaly	unvaccinated	112,003	382(0.34)		Baseline		
	vaccinated	17,485	64(0.37)	1.07(0.82 to 1.39)	0.60	1.01(0.76 to 1.33)	0.94
Stillbirth	unvaccinated	112,003	815(0.73)		Baseline		
	vaccinated	17,485	90(0.51)	0.70(0.56 to 0.87)	0.002	0.60(0.47 to 0.76)	<0.001
Intrauterine growth restriction	unvaccinated	112,003	2,169(1.94)		Baseline		
	vaccinated	17,485	347(1.98)	.02(0.91 to 1.14)	0.66	1.04 (0.92 to 1.18)	0.44
Low birth weight	unvaccinated	112,003	7,007(6.2)		Baseline		
	vaccinated	17,485	1,017(5.8)	0.92(0.86 to 0.99)	0.02	1.05(0.96 to 1.14)	0.27
Very low birth weight	unvaccinated	112,003	1,468(1.3)		Baseline		
	vaccinated	17,485	145(0.8)	0.62(0.53 to 0.74)	<0.001	1.01(0.76 to 1.34)	0.90
NICU admission	unvaccinated	112,003	11,347(10.1)		Baseline		
	vaccinated	17,485	1,762(10.0)	0.99(0.94 to 1.04)	0.82	1.00(0.94 to 1.06)	0.80
Low 5-minute Apgar score	unvaccinated	112,003	1,341(1.2)		Baseline		
	vaccinated	17,485	153(0.8)	0.72(0.61 to 0.86)	<0.001	0.98(0.78 to 1.23)	0.88
Neonatal death	unvaccinated	112,003	146(0.13)		Baseline		
	vaccinated	17,485	15(0.09)	0,65(0.38 to 1.11)	0.12	1.07(0.60 to 1.91)	0.80

^a^preterm birth, extremely preterm birth, congenital anomalies, stillbirth, and IUGR are adjusted for maternal age, maternal underlying medical conditions, smoking or other addictions in pregnant mothers, maternal educational level, prepregnancy body mass individuals, and parity

LBW, VLBW, NICU admission, low 5-minute Apgar score, and neonatal death are adjusted for gestational age at birth, maternal age, maternal underlying medical conditions, smoking or other addictions in pregnant mother, maternal educational level, prepregnancy body mass individuals and parity.

**Table 3:** Crude and adjusted odds ratios of adverse perinatal outcomes according to COVID-19 vaccination doses

**Adverse pregnancy outcome**	**Vaccination status**	**No.**	**No of cases (%)**	**Unadjusted odds ratio (95%CI)**	**Unadjusted *P* value**	**adjusted odds ratio (95%CI)**^a^	**Adjusted *p* value**
Preterm birth	unvaccinated	112,003	9,151(8.17)		Baseline		
	1 dose	6,774	589(8.70)	1.07(0.98 to 1.16)	0.12	1.00(0.91 to 1.10)	0.89
	2 doses	10,711	807(7.53)	0.91(0.84 to 0.98)	0.02	0.85(0.79 to 0.92)	<0.001
Extremely preterm birth	unvaccinated	112,003	1,497(1.34)		Baseline		
	1 dose	6,774	70(1.03)	0.77(0.60 to 0.98)	0.03	0.73(0.56 to 0.95)	0.01
	2 doses	10,711	63(0.59)	0.43(0.33 to 0.56)	<0.001	0.43(0.33 to 0.56)	<0.001
Congenital anomaly	unvaccinated	112,003	382(0.34)		Baseline		
	1 dose	6,774	19(0.28)	0.82(0.51 to 1.30)	0.40	0.76(0.46 to 1.24)	0.27
	2 doses	10,711	45(0.42)	1.23(0.90 to 1.68)	0.18	1.16(0.84 to 1.61)	0.35
stillbirth	unvaccinated	112,003	815(0.73)		Baseline		
	1 dose	6,774	45(0.66)	0.91(0.67 to 1.23)	0.55	0.74(0.53 to 1.04)	0.08
	2 doses	10,711	45(0.42)	0.57(0.42 to 0.77)	<0.001	0.50(0.36 to 0.70)	<0.001
Intrauterine growth restriction	unvaccinated	112,003	2,169(1.94)		Baseline		
	1 dose	6,774	135(1.99)	1.02(0.86 to 1.22)	0.74	`.07 (0.88 to 1.29)	0.47
	2 doses	10,711	212(1.98)	1.02(0.88 to 1.17)	0.75	1.03(0.88 to 1.20)	0.64
Low birth weight	unvaccinated	112,003	7,007(6.26)		Baseline		
	1 dose	6,774	416(6.14)	0.98(0.88 to 1.08)	0.70	0.99(0.86 to 1.14)	0.92
	2 doses	10,711	601(5,61)	0.89(0.81 to 0.97)	0.008	1.08(0.97 to 1.21)	0.13
Very low birth weight	unvaccinated	112,003	1,468(1.31)		Baseline		
	1 dose	6,774	73(1.08)	0.82(0.64 to 1.03)	0.10	1.04(0.69 to 1.57)	0.84
	2 doses	10,711	72(0.67)	0.50(0.40 to 0.64)	<0.001	0.99(0.69 to 1.43)	0.99
NICU admission	unvaccinated	112,003	11,347(10.13)		Baseline		
	1 dose	6,774	725(10.70)	1.06(0.98 to 1.15)	0.13	1.02(0.93 to 1.11)	0.65
	2 doses	10,711	1,037(9.68)	0.95(0.88 to 1.01)	0.14	0.99(0.92 to 1.07)	0.98
Low 5-minute Apgar score	unvaccinated	112,003	1,341(1.20)		Baseline		
	1 dose	6,774	69(1.02)	0.84(0.66 to 1.08)	0.18	0.87(0.62 to 1.24)	0.46
	2 doses	10,711	84(0.78)	0.65(0.52 to 0.81)	<0.001	1.06(0.80 to 1.40)	0.67
Neonatal death	unvaccinated	112,003	146(0.13)		Baseline		
	1 dose	6,774	5(0.07)	0,56(0.23 to 1.38)	0.21	0.62(0.22 to 1.74)	0.36
	2 doses	10,711	10(0.09)	0.71(0.37 to 1.35)	0.30	1.40(0.76 to 2.94)	0.24

^a^preterm birth, extremely preterm birth, congenital anomalies, stillbirth, and IUGR are adjusted for maternal age, maternal underlying medical conditions, smoking or other addictions in pregnant mothers, maternal educational level, prepregnancy body mass individuals, and parity

LBW, VLBW, NICU admission, low 5-minute Apgar score, and neonatal death are adjusted for gestational age at birth, maternal age, maternal underlying medical conditions, smoking or other addictions in pregnant mother, maternal educational level, prepregnancy body mass individuals and parity

**Table 4:** Crude and adjusted Odds ratios for adverse perinatal outcomes according to COVID-19 vaccination trimester

**Adverse pregnancy outcome**	**Vaccination status**	**No**	**No of cases (%)**	**Unadjusted odds ratio (95%CI)**	**Unadjusted *P* value**	**adjusted odds ratio (95%CI)**^a^	**Adjusted *p* value**
Preterm birth	unvaccinated	112,003	9,151(8.17)		Baseline		
	First trimester	1,202	134(11.15)	1.41(1.17 to 1.68)	<0.001	1.27(1.04 to 1.55)	0.01
	Second trimester	10,250	896(8.74)	1.07(1.00 to 1.15)	0.04	1.00(0.93 to 1.08)	0.85
	Third trimester	6,033	366(6.07)	0.72(0.65 to 0.80)	<0.001	0.68(0.61 to 0.77)	<0.001
Extremely preterm birth	unvaccinated	112,003	1,497(1.34)		Baseline		
	First trimester	1,202	13(1.08)	0.80(0.46 to 1.39)	0.44	0.77(0.43 to 1.38)	0.39
	Second trimester	10,250	105(1.02)	0.76(0.62 to 0.93)	0.008	0.72(0.58 to 0.90)	0.004
	Third trimester	6,033	15(0.25)	0.18(0.11 to 0.30)	<0.001	0.20(0.12 to 0.33)	<0.001
Congenital anomaly	unvaccinated	112,003	382(0.34)		Baseline		
	First trimester	1,202	6(0.50)	1.46(0.65 to 3.29)	0.35	1.50(0.66 to 3.38)	0.32
	Second trimester	10,250	45(0.44)	1.28(0.94 to 1.75)	0.10	1.21(0.87 to 1.68)	0.23
	Third trimester	6,033	13(0.22)	0.63(0.36 to 1.09)	0.10	0.55(0.30 to 1.01)	0.05
stillbirth	unvaccinated	112,003	815(0.73)		Baseline		
	First trimester	1,202	10(0.83)	1,14(0.61 to 2.14)	0.67	1,09(0.58 to 2.06)	0.76
	Second trimester	10,250	64(0.62)	0.85(0.66 to 1.10)	0.23	0.74(0.56 to 0.98)	0.03
	Third trimester	6,033	16(0.27)	0.36(0.22 to 0.59)	<0.001	0.25(0.13 to 0.46)	<0.001
Intrauterine growth restriction	unvaccinated	112,003	2,169(1.94)		Baseline		
	First trimester	1,202	30(2.50)	1,29(0.89 to 1.86)	0.16	1.37(0.93 to 2.02)	0.10
	Second trimester	10,250	202(1.97)	1.01(0.88 to 1.17)	0.81	1.00(0.86 to 1.17)	0.91
	Third trimester	6,033	115(1.91)	0.98(0.81 to 1.18)	0.86	1.05(0.86 to 1.28)	0.59
Low birth weight	unvaccinated	112,003	7,007(6.26)		Baseline		
	First trimester	1,202	98(8.15)	1.33(1.08 to 1.63)	0.007	1.27(0.96 to 1.67)	0.09
	Second trimester	10,250	639(6.23)	0.99(0.91 to 1.08)	0.93	0.99(0.88 to 1.11)	0.89
	Third trimester	6,033	280(4.64)	0.72(0.64 to 0.82)	0.001	1.11(0.96 to 1.28)	0.15
Very low birth weight	unvaccinated	112,003	1,468(1.31)		Baseline		
	First trimester	1,202	17(1.41)	1.08(0.66 to 1.74)	0.75	1.80(0.82 to 3.92)	0.13
	Second trimester	10,250	106(1.03)	0.78(0.64 to 0.95)	0.01	0.90(0.64 to 1.28)	0.57
	Third trimester	6,033	22(0.36)	0.27(0.18 to 0.42)	<0.001	1.10(0.65 to 1.87)	0.70
NICU admission	unvaccinated	112,003	11,348(10.13)		Baseline		
	First trimester	1,202	148(12.31)	1.24(1.04 to 1.48)	0.01	1.15(0.94 to 1.40)	0.15
	Second trimester	10,250	1,057(10.31)	1.01(0.95 to 1.09)	0.56	1.01(0.94 to 1.09)	0.62
	Third trimester	6,033	557(9.23)	0.90(0.82 to 0.98)	0.02	0.95(0.86 to 1.05)	0.38
Low 5-minute Apgar score	unvaccinated	112,003	1,341(1.20)		Baseline		
	First trimester	1,202	15(1.25)	1.04(0.62 to 1.74)	0.87	1.04(0.50 to 2.16)	0.90
	Second trimester	10,250	110(1.07)	0.89(0.73 to 1.08)	0.26	1.04(0.80 to 1.36)	0.73
	Third trimester	6,033	28(0.46)	0.38(0.26 to 0.55)	<0.001	0.80(0.51 to 1.27)	0.36
Neonatal death	unvaccinated	112,003	146(0.13)		Baseline		
	First trimester	1,202	2(0.17)	1.27(0.31 to 5.1)	0.73	1.34(0.29 to 6.03)	0.70
	Second trimester	10,250	11(0.11)	0.82(0.44 to 1.51)	0.53	1.25(0.66 to 2.39)	0.48
	Third trimester	6,033	2(0.03)	0.25(0.06 to 1.02)	0.05	0.36(0.04 to 2.65)	0.31

^a^preterm birth, extremely preterm birth, congenital anomalies, stillbirth, and IUGR are adjusted for maternal age, maternal underlying medical conditions, smoking or other addictions in pregnant mothers, maternal educational level, prepregnancy body mass individuals, and parity

LBW, VLBW, NICU admission, low 5-minute Apgar score, and neonatal death are adjusted for gestational age at birth, maternal age, maternal underlying medical conditions, smoking or other addictions in pregnant mother, maternal educational level, prepregnancy body mass individuals and parity.

Extremely preterm birth included 1.26% of all births (0.76% between vaccinated individuals vs 1.34% between unvaccinated individuals). Adjusted analysis indicated a statistically significant decrease in the incidence of extremely preterm birth in individuals vaccinated against SARS-CoV-2 during pregnancy (*P* =<0.001, aOR=0.55, [95% CI, 0.45 to 0.66]) (Table 2). The protective effect was observed in individuals vaccinated with either 1 or 2 doses (Table 3). Comparing the trimester of pregnancy while vaccinated, there was no significant increase in the incidence of extremely preterm birth associated with vaccination during the first trimester. However, a significant decrease was seen in the risk of extremely preterm birth in those vaccinated during the second and third trimesters (Table 4).

Stillbirth occurred in 0.70% of births (0.51% between vaccinated individuals vs 0.73% between unvaccinated individuals). According to the adjusted analysis, a significant reduction in stillbirths was found among vaccinated individuals (*P* =<0.001, aOR=0.60, [95% CI, 0.47 to 0.76]) (Table 2). At least receiving 2 doses during pregnancy was required to observe a decrease in stillbirth (Table 3). First-trimester vaccination caused no statistically significant increase in the incidence of stillbirth. However, adjusted analysis indicated a decrease in stillbirth in individuals vaccinated during the second and third trimesters of pregnancy (Table 4).

Unadjusted analysis indicated a decrease in the incidence of LBW, VLBW, and low 5-minute Apgar score. After adjustment for gestational age, this protective effect was not observed. This means there is no difference in the risk of these outcomes in exposed and unexposed neonates with the same gestational age.

Adjusted analysis indicated no statistically significant increase in the risk of congenital anomalies, intrauterine growth restriction, neonatal intensive care unit admission and neonatal death between neonates born from vaccinated and unvaccinated individuals (Table 2). The results were similar for vaccination with 1 or 2 doses (Table 3) and vaccination during all trimesters (Table 4).

## Discussion

This large retrospective cohort study aimed to compare the incidence of adverse perinatal outcomes between individuals vaccinated against SARS-CoV-2 with the Sinopharm vaccine and unvaccinated individuals in Tehran, Iran.

Sinopharm vaccination during the third trimester of pregnancy decreased the incidence of preterm birth. Also, extremely preterm birth declined significantly in those vaccinated during the second and third trimesters. Although at least 2 doses of Sinopharm vaccination were required for a decrease in the risk of preterm birth, only 1 dose can lower the incidence of extremely preterm birth. Similar studies conducted on mRNA vaccines reported no statistically significant increase in the incidence of preterm birth and extremely preterm birth between the vaccinated and unvaccinated groups [15, 20, 21]. Recent studies indicated an association between third-trimester COVID-19 vaccination and lower risk of preterm birth [21]

Nonetheless, a slight increase in preterm birth was observed in individuals vaccinated during the first trimester of pregnancy. This increased risk could be caused by early vaccination. However, such a deduction requires further studies. Articles conducted on mRNA vaccines reported no increase in the risk of preterm birth in the Case of Maternal first-trimester vaccination [16].

The incidence of stillbirth was significantly lower in individuals who received the Sinopharm vaccine while pregnant. At least 2 doses of Sinopharm vaccination during pregnancy were required to observe the protective effects against stillbirth. Similar protective effects were reported in studies conducted on mRNA vaccines [17, 22].

The adjusted analysis indicated a lower risk of preterm birth, extremely preterm birth, and stillbirth. The results can be related to a lower risk of SARS-CoV-2 infection in the vaccinated individuals or antibody transportation to the fetus. The third-trimester vaccination is associated with higher IgG antibody levels in the fetus. Also, Vaccination with 2 doses is associated with higher IgG antibodies in the fetus blood sample compared to vaccination with only 1 dose [23, 24]

There was no statistically significant increase in the risk of IUGR, low 5-minute Apgar score, NICU admission, congenital anomaly, LBW, VLBW, and neonatal death between individuals vaccinated with the Sinopharm vaccine during pregnancy and unvaccinated mothers. Existing articles on the use of mRNA vaccines during pregnancy indicated an association between vaccination and a decreased risk of low 5-minute Apgar score and NICU admission [21, 25] Existing articles indicated no association between vaccination and an increased risk of perinatal outcomes [25, 26].

To our knowledge, this is the first study about adverse perinatal outcomes in individuals vaccinated with the Sinopharm vaccine, which is the main strength of this study. Other strengths are coverage of individuals vaccinated during the first trimester and the population-based setting that led to reduced risk of selection bias.

The following study limitations should be noted. First, data on SARS-CoV-2 infection during pregnancy was unavailable in our study. Exclusion of those infected during pregnancy could lead to more accurate results on the effect of Sinopharm vaccination on perinatal outcomes. Second, although 17,485 mothers were exposed to vaccination during pregnancy, only 1,202 were vaccinated during the first trimester; therefore, the number of newborns exposed in the first trimester is underpowered to detect the incidence of rare adverse perinatal outcomes. Third, although the analysis adjusted for a broad range of potential confounding factors, adjustment could have been conducted for other potential covariates (like household income) if the data was available.

Pregnant women are at risk of severe COVID-19 and related complications [1, 2, 27]. Maternal COVID-19 vaccination during pregnancy reduces COVID-19 complications in infected pregnant individuals [28]. Also, immunization against SARS-CoV-2 during pregnancy has the extra benefit of transferring antibodies to the developing fetus, which can protect the infant against COVID-19 infection for a few months [29, 24].

The results of this study support the safety of vaccination with the Sinopharm vaccine during the second and third trimester of pregnancy. The findings of this study can help healthcare practitioners and policymakers educate pregnant women about the safety and benefits of vaccinations for both themselves and their developing fetuses.

Further studies must focus on the incidence of perinatal outcomes in first-trimester vaccination with the Sinopharm vaccine. Also, the incidence of other perinatal outcomes like miscarriage can be a subject of future studies.

## Conclusion

The finding of this study indicated no adverse pregnancy outcome due to vaccination with the Sinopharm vaccine during the second and third trimesters of pregnancy. The overall risk of adverse perinatal outcomes was lower in vaccinated individuals compared to the unvaccinated group. Regarding increased risk of preterm birth related to First-trimester vaccination in this study, further studies are required.

## Data Availability

Data for this study was obtained from the Ministry of Health and Medical Education of Iran but restrictions apply to public availability of these data. Data could be available upon reasonable request from the neonatal health office, Ministry of Health and Medical Education via email at habibelahi@health.gov.ir

## Abbreviations

IUMS: Iran University of Medical Sciences
LBW: Low Birth Weight
VLBW: Very low Birth Weight
NICU: Neonatal Intensive Care Unit
